# Protocol for a multicenter observational study on fatigue across physical–mental conditions within the German center for mental health

**DOI:** 10.3389/fpsyt.2026.1774114

**Published:** 2026-04-10

**Authors:** R. Erschens, S. Zipfel, M. Bentele, S. H. Adam, C. Schröpel, H. Heitmann, M. Binneböse, V. Zimmermann-Schlegel, J. Reichert, C. Rometsch, I. Ottlewski, A. Schappert, H.-C. Friederich, H. Gündel, M. Rose, J. Tesarz, F. Junne, P. Henningsen, A. Stengel

**Affiliations:** 1 Department of Psychosomatic Medicine and Psychotherapy, University Hospital Tübingen, Tübingen, Baden-Württemberg, Germany; 2 German Center for Mental Health (DZPG), Tübingen, Germany; 3 Klinikum rechts der Isar, Department and Outpatient Clinic of Psychosomatic Medicine and Psychotherapy, Technical University of Munich, Munich, Bavaria, Germany; 4 German Center for Mental Health (DZPG), Munich, Germany; 5 Department of Psychosomatic Medicine and Psychotherapy, University Hospital Magdeburg, Magdeburg, Saxony-Anhalt, Germany; 6 German Center for Mental Health (DZPG), Magdeburg, Germany; 7 Department of General Internal Medicine and Psychosomatics, University Hospital Heidelberg, Heidelberg, Baden-Württemberg, Germany; 8 German Center for Mental Health (DZPG), Heidelberg, Germany; 9 Department and Outpatient Clinic of Psychosomatic Medicine and Psychotherapy, University Medical Center Mainz, Mainz, Rhineland-Palatinate, Germany; 10 Department of Psychosomatic Medicine, Charité – Universitätsmedizin Berlin, Berlin, Germany; 11 German Center for Mental Health (DZPG), Berlin, Germany; 12 Department of Psychosomatic Medicine and Psychotherapy, University Hospital Ulm, Ulm, Baden-Württemberg, Germany; 13 German Center for Mental Health (DZPG), Ulm, Germany; 14 Clinic of Psychosomatic Medicine and Psychotherapy, Klinikum Stuttgart, Stuttgart, Baden-Württemberg, Germany

**Keywords:** biopsychosocial factors, fatigue, persistent physical symptoms, psychosomatic medicine, transdiagnostic psychopathology

## Abstract

**Introduction:**

Fatigue is a common and debilitating symptom across a wide range of chronic physical and mental health conditions. It affects physical, cognitive, and emotional functioning and substantially reduces quality of life. Despite its clinical relevance, fatigue research remains largely diagnosis-specific, resulting in limited understanding of cross-diagnostic patterns and the interplay between somatic, psychological, and psychosocial factors. Comparative data spanning different physical and mental health conditions remain limited.

**Objectives:**

This multicentre, cross-sectional observational study is designed to characterise the multidimensional profile of fatigue across seven diagnostic cohorts representing metabolic, inflammatory, oncological, neuroimmunological, post-infectious, and functional-psychosomatic conditions included in the framework of the German Center for Mental Health.

**Methods and analysis:**

Participants will complete a standardised psychometric assessment battery assessing fatigue severity, post-exertional malaise, physical functioning, and related mental-health and psychosocial variables. Key domains include depressive and anxiety symptoms, somatisation, trauma history, pain, work ability, and occupational stress. Data will be analysed descriptively and comparatively to examine shared versus condition-specific fatigue patterns within a bio-psycho-social framework.

**Ethics and dissemination:**

Ethical approval has been obtained from all participating centres prior to the conduct of the study procedures described herein, and the study is registered in the German Clinical Trials Register (DRKS00037687). Findings will be disseminated through peer-reviewed publications, conference presentations, and stakeholder networks of the German Center for Mental Health.

## Introduction

Fatigue is a common and distressing symptom affecting approximately 20% of the general adult population and constituting one of the most frequent reasons for primary care consultation (1, 2). Fatigue represents one of the most frequent manifestations of persistent physical symptoms (PPS), defined as distressing somatic symptoms that may persist for months or longer across a wide range of medical and psychosocial contexts and disorders.

Regardless of their initial cause, persistent physical symptoms (PPS) are shaped by a dynamic and mutually reinforcing interplay of biological, psychological, and social mechanisms. These include alterations in central nervous system processing (e.g. symptom perception and regulation), learning and expectation effects, affective and cognitive factors such as threat appraisal and illness beliefs, as well as social and healthcare-related influences (3). Importantly, PPS are associated with substantial functional impairment, reduced quality of life, high healthcare utilization, and unmet treatment needs across healthcare systems, despite often limited explanatory power of conventional disease markers (4).

Clinically, fatigue is characterized by a persistent reduction in energy or an increased need for rest over several weeks, often accompanied by additional symptoms such as cognitive dysfunction or post-exertional malaise (5). These expressions mirror complex interactions among physiological, cognitive, and affective processes, illustrating what may be termed the physical–mental interplay underlying fatigue. Beyond this descriptive definition, fatigue is widely understood as a multidimensional phenomenon comprising physical, cognitive, and emotional components. Berrios’ tripartite model (6) provides a useful conceptual starting point; however, these dimensions have not been systematically compared across diagnostic boundaries. In the context of the present study, the model therefore serves as a theoretical anchor for empirically examining how fatigue manifests across different physical–mental health conditions.

Fatigue is highly prevalent and burdensome across numerous disorders, including cancer, multiple sclerosis, inflammatory bowel disease, patients receiving hemodialysis as well as mental health disorders such as depression and ADHD (7–12). It is also among the most persistent symptoms following SARS-CoV-2 infection, including post-COVID conditions (13, 14). Moreover, post-COVID conditions are likely to increase the prevalence of Myalgic Encephalomyelitis/Chronic Fatigue Syndrome (ME/CFS), which are also characterized by chronic fatigue and post-exertional symptom exacerbation (15).

While disease-specific studies demonstrate its clinical relevance, most research remains siloed within individual diagnoses. Emerging evidence suggests that fatigue transcends disorder categories, affects 27–55% of individuals across chronic diseases, and exhibits shared phenomenological patterns such as progressive energy depletion (16, 17). Qualitative syntheses show consistent phenomenological patterns such as “running out of batteries” and progressive energy loss across somatic and psychosomatic conditions (17).

This underscores the need for integrative clinical frameworks and treatment approaches on fatigue symptoms. Thus, focusing on transdiagnostic approaches to fatigue; that is, approaches that target mechanisms and symptom patterns shared across disorders rather than being specific to a single diagnosis; aims to identify common processes that cut across diagnostic boundaries. Recent research has begun to frame fatigue as a transdiagnostic symptom across diagnostic boundaries. For example, the IDEA-FAST project investigates fatigue in neurodegenerative and immune-mediated inflammatory disorders using wearable devices and repeated patient-reported outcomes (PROs) to explore associations between subjective fatigue and objective gait characteristics (18). Similarly, a recent study employs digital health technologies, ecological momentary assessment (EMA), and qualitative methods to examine fatigue experiences over time in individuals with myeloma, long COVID, and heart failure (19). While these studies represent important methodological advances, particularly in capturing temporal variability and demonstrating the feasibility of digital approaches, comparative data based on standardized psychometric assessments across a broader range of physical conditions remain limited.

**Figure 1:**
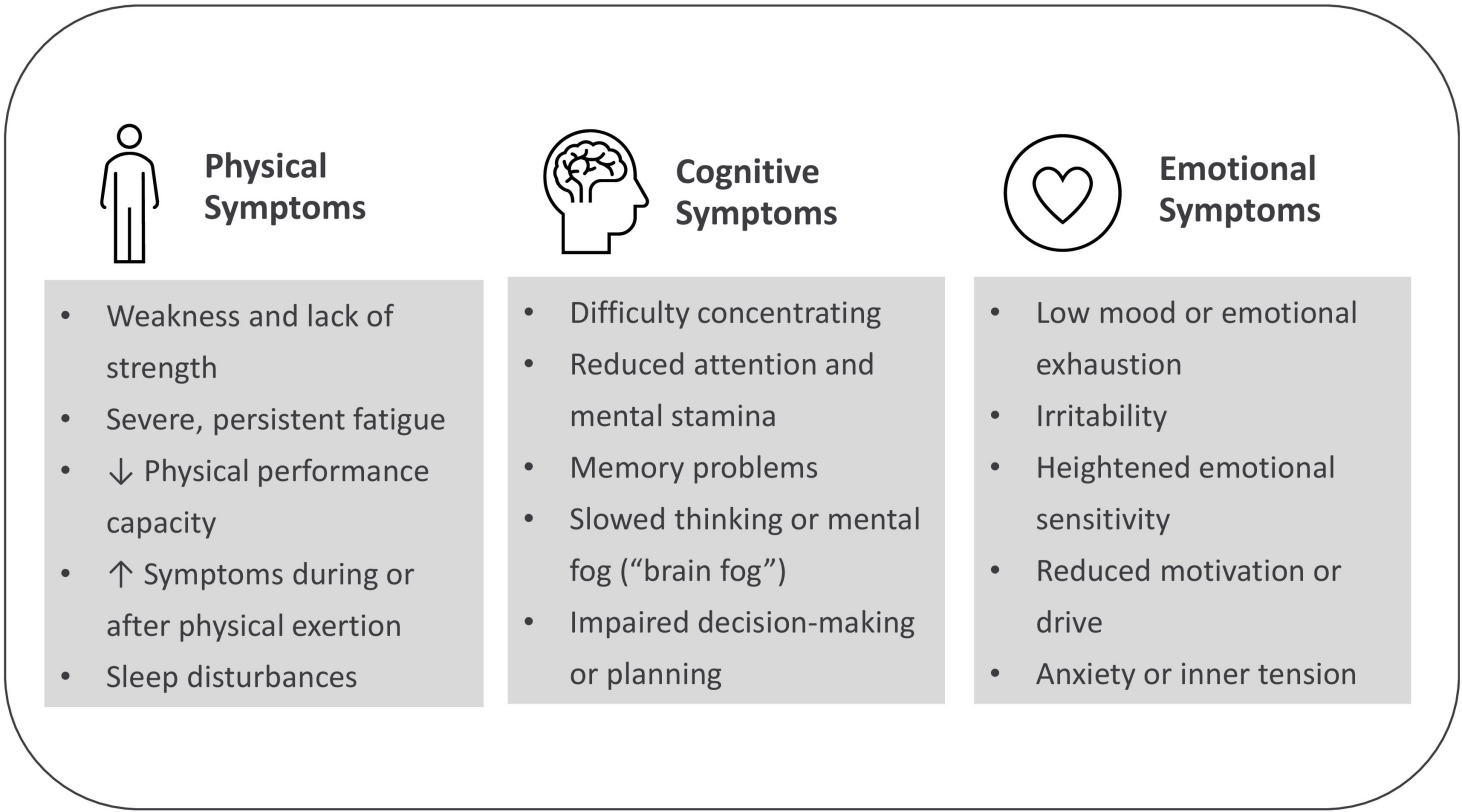
Symptom dimensions of fatigue according to Berrios (6). **Figure 1** Depicts the three core symptom domains of fatigue—physical, cognitive, and emotional. Physical symptoms involve reduced energy and performance; cognitive symptoms include concentration and memory difficulties (“brain fog”); emotional symptoms encompass low mood, irritability, and reduced motivation. This tripartite model follows Berrios (6) and serves as the conceptual basis for the multidimensional assessment in the present study.

Network studies further highlight fatigue as a central node linking somatic and mental health symptoms, indicating common underlying mechanisms and supporting a transdiagnostic research approach (20). Scoping reviews indicate that fatigue may manifest largely independently of the underlying medical diagnosis (21) and recent network analyses identifying fatigue as a central symptom linking diverse disorders and thus a potential key target for intervention (20). A conceptual overview of this rationale is depicted in **Figure 1**, outlining how transdiagnostic symtoms across the physical, cognitive and emotional domain converge into the symptom experience of fatigue. Taken together, these observations emphasise the need for cross-diagnostic, multidimensional assessment of fatigue that integrates somatic and psychological dimensions.

## Objectives

The present multicentre cross-sectional study is designed to address gaps in the understanding of transdiagnostic fatigue features and their associations with psychosocial and somatic factors by examining fatigue profiles across seven chronic physical–mental conditions. Specifically, the study is intended to characterise fatigue across its physical, cognitive, and emotional dimensions and to examine shared versus condition-specific associations with psychological, somatic, and psychosocial factors, thereby informing future research on tailored and mechanism-based intervention approaches. By systematically assessing fatigue across cohorts within a unified framework, the present study builds on established conceptual models such as Berrios’ tripartite structure (6) enabling empirical comparison of e.g. physical, cognitive, and emotional fatigue dimensions across diverse conditions.

### Specific aims

- To characterise the multidimensional symptom profile of fatigue across diagnostic groups using a core set of validated self-report instruments (see **Table 1**).
- To examine associations between fatigue and psychological and psychosocial variables, including depressive and anxiety symptoms, somatisation, distress, trauma exposure, and quality of life.
- To explore contextual and occupational correlates of fatigue, such as pain, work ability, effort–reward balance, and psychosocial safety climate.
- To compare fatigue profiles between diagnostic cohorts to examine common versus condition-specific patterns of symptom expression and their potential relevance for future research.
- To examine psychological, behavioural, and systemic factors associated with fatigue across physical–mental conditions.

**Table 1:** Overview on the core set of fatigue instruments implemented in the study.

Questionnaire	Description	No. of items	Subscales	Response format	Reliability	Validity	Scores/Cut-off values
Fatigue Severity Scale (FSS) (22, 23)	Measurement of fatigue.	9	n.a.	1(not true at all)-7 (completely true)	Cronbach’s α = 0.94 (overall sample) (22); Re-Test-Reliability (2 weeks): *r* = 0.63-0.88 (MS patients), *r* = 0.54-0.84 (control group) (22)	Convergent validity (with VAS): *r* = 0.6-0.73 (MS patients), *r* = 0.33-0.37 (control group) (22)	Overall score: 1-7; Cut-off values: ≥ 4 fatigue, <4 non-fatigue
Visual analogue scale(VAS) (22)	Measures the level of fatigue. The VAS forms part of the FSS and was employed in the validation paper for the FSS to verify its convergent validity.	1	n.a.	0 (no fatigue)-10 (most severe fatigue)	Re-test reliability: *r* = 0.69-0.73 (22)	n.a.	Overall score: 1-10
Chalder Fatigue Scale (CFQ-11) (24, 25)	Measures severity of physical and mental fatigue.	11	Physical fatigue (items 1-7), psychological fatigue (items 8-11)	0 = less than usual, 1 = no more than usual, 2 = worse than usual, 3 = much worse than usual	Cronbach’s α = 0.89 (24)	n.a.	2 analysis methods (26); (1) binary Scoring Method: score 0-11, <4 no fatigue, ≥4 severe fatigue, (2) likert scoring method: overall score 0-33
DePaul Symptom Questionnaire Post-Exertional Malaise (DSQ-PEM) (27, 28)	Measurement of post-exertional malaise (PEM). Operationalization through retrospective assessment (last 6 months).	15	Frequency (items 1a-5a), Severity (items 1b-5b); additional items: PEM duration (items 7-9), Quick recovery (item 6), Exercise exacerbation (item 10)	Frequency: 0 = none of the time, 1 = a little of the time, 2 = about half the time, 3 = most of the time, 4 = all of the time; Severity:0 = symptom not present, 1 = mild, 2 = moderate, 3 = severe, 4 = very severe; additional items: binary (yes/no), PEM duration in hours	Available for the first 5 items (28): Cronbach’s α = .94 (general population sample), Cronbach’s α = .91 (Post covid sample)	PEM total score (28): Convergent validity (with PHQ-4): *r* = .599 (general population sample); *r* = .471 (Post covid sample)	Continous PEM scores: 0-8 (for the first five items), Binary PEM scores (yes/no) (28): yes = (frequency and severity ≥2) AND (item7 OR item8 = 1) AND (item 9 <14h), Items 6 and 10 provide further information/description
PROMIS Fatigue Short Form (PROMIS SF) (29–32), 7-item customized short form	Measurement of fatigue.	7	n.a.	Intensity: 1 = not at all, 2 = a little bit, 3 = somewhat, 4 = quite a bit, 5 = very much; Frequency: 1 = never, 2 = rarely, 3 = sometimes, 4 = often, 5 = always	n.a.	n.a.	Sum scores (7-35) are transformed to a T-score metric
Systemic Exertion Intolerance Disease (SEID) criteria of the Institute of Medicine (IOM) (33–35)	IOM criteria for the diagnosis of ME/CFS.	5	n.a.	binary (yes/no)	n.a.	n.a.	Binary score: suspected diagnosis of ME/CFS yes: first three criteria AND (cognitive impairment OR orthostatic intolerance)
Short Form Health Survey (SF-36) – Subscale physical functioning (36–38)	Measures physical functioning in relation to everyday activities and mobility.	10	n.a.	1 = yes, very limited, 2 = yes, somewhat restricted, 3= no, not limited at all	Cronbach’s α = .73-.92 (37)	Convergent validity (with NHP-physical activity): *r* = 0.43 (students), *r* = 0.78 (backpain patients) (37)	Sum score (10-30) is transformed into a scale ranging from 0 (worst quality of life) to 100 (best quality of life) (39)

n.a., not applicable.

## Methods and analysis

### Study design and data collection

This study is designed as a multicentre, cross-sectional investigation with prospective recruitment and ongoing data collection. Data are being collected at clinical sites within the German Center for Mental Health (DZPG), including the university hospitals of Tübingen, Heidelberg, Munich (Technical University of Munich), Magdeburg, and Berlin (Charité). Participant recruitment commenced in spring 2024 and is ongoing across all participating sites.

Participants are invited to complete an online survey assessing fatigue and related psychosocial and clinical variables. Data are collected and managed using REDCap (Research Electronic Data Capture) hosted at the University Hospital Tübingen, providing secure data entry, audit trails, validated workflows, and automated export to statistical software (40, 41). Participants access the digital assessment battery via personalised links. After completion of the questionnaires, participants receive compensation in the form of a voucher for a nationwide bookstore.

### Sociodemographic and clinical variables

All participants are invited to complete a standardised psychometric assessment battery covering the physical, cognitive, and emotional dimensions of fatigue, together with related mental health and psychosocial variables. The assessment battery comprises a comprehensive set of validated instruments and requires approximately 45 minutes to complete. To minimise participant burden, particularly in individuals experiencing pronounced fatigue, the online survey is designed to allow flexible completion, including the option to pause and resume the assessment, and participants may complete the questionnaires at their own pace. Measures include fatigue severity, post-exertional malaise, quality of life, psychological distress, and occupational functioning.

Participants provide sociodemographic and medical information including age, sex, nationality, marital status, education, employment status, household composition, and income. Occupational characteristics such as working hours, shift work, and company size are recorded alongside lifestyle factors including smoking, alcohol consumption, drug use, pregnancy, and recent stress exposure. Medical history and current diagnoses are assessed using a checklist of major chronic physical and mental health conditions (e.g., multiple sclerosis, cancer, chronic kidney disease or inflammatory bowel disease, post-COVID syndrome). Where available and where consent has been provided, these self-reported data are complemented by information extracted from patients’ clinical records.

Additional items capture prior psychotherapeutic or psychiatric treatment, periods of incapacity for work, and experiences of occupational reintegration following illness. These variables are used to characterise the study population, address potential confounding, and support descriptive and comparative analyses across diagnostic cohorts.

### Fatigue measures

Fatigue is assessed across its physical, cognitive, and emotional dimensions using validated psychometric instruments, including the Fatigue Severity Scale (FSS) (22, 23), Chalder Fatigue Scale (24, 25), DePaul Symptom Questionnaire – Post-Exertional Malaise subscale (27, 28), Patient-Reported Outcomes Measurement Information System (PROMIS Fatigue) (29–32), Systemic Exertion Intolerance Disease (SEID) criteria of the Institute of Medicine (33–35), and the Short Form-12 Physical Functioning subscale (SF-12 PF) (36–38). Together, these instruments capture complementary aspects of fatigue, including general fatigue severity, post-exertional malaise, and functional impairment. A comprehensive overview of all fatigue-related instruments, including theoretical background, operationalisation, response formats, scoring, and psychometric properties, is provided in **Table 1**.

### Comorbid variables

To contextualise fatigue within the broader bio-psycho-social framework, additional validated self-report measures assess comorbid mental distress, quality of life, pain, and work-related factors. These include depressive symptoms (Patient Health Questionnaire, PHQ-9 (42)), anxiety (Generalised Anxiety Disorder Scale, GAD-7 (43)), somatisation (PHQ-15 (44)), psychological responses to bodily symptoms (SSD-12 (45)), trauma exposure (Primary Care PTSD Screen, PC-PTSD-5 (46), and Childhood Trauma Questionnaire, CTQ (47)), and health-related quality of life (Short Form Health Survey, SF-12 (48, 49)). Pain and functional symptoms are assessed using the Numerical Rating Scale (NRS-11 (50)) and the Widespread Pain Index (WPI (51)), while occupational functioning and psychosocial context are measured with the Work Ability Index (WAI (52)), the Effort–Reward Imbalance Short Form (ERI-SF (53)), and the Psychosocial Safety Climate Scale (PSC-4 (54)). The PECAN (Perceived Causal Network) (55–57) method captures participants’ subjective perceptions of directed relationships between symptoms previously defined by the research team. These ten symptoms reflect fatigue, post-exertional malaise, and other transdiagnostically relevant psychosomatic features. Based on these ten symptoms, participants create up to 90 directed causal ratings (10 × 9), each scored on a 0–10 scale. A complete overview of all psychometric instruments, operationalisation, and psychometric properties, is provided in **Table 2**.

**Table 2:** Overview on the instruments used to measure the secondary outcomes of the study. .

Phenomenology	Questionnaire	No. of items	Subscales	Reliability	Validity
Depression	Patient Health Questionnaire (PHQ-9) (42, 58)	9	n.a.	Cronbach’s α = 0.86-0.89 (42)	Criterion validity (42): AUC = 0.95
Somatization/Somatic symptoms	Patient Health Questionnaire (PHQ-15) (44)	15	n.a.	Cronbach’s α = 0.80 (44)	High construct validity (44)
Psychological response to bodily symptoms	Somatic Symptom Disorder (SSD-12) (45, 59)	12	cognitive, affective, and behavioural aspects	Cronbach’s α = 0.95 (45)	Construct validity with PHQ-15 (45): *r* = 0.47
Anxiety	Generalized Anxiety Disorder (GAD-7) (43)	7	n.a.	Cronbach’s α = 0.92; test-retest reliability = 0.83 (43)	Convergent validity with Beck Anxiety Inventory (43): *r* = 0.72, with Symptom Checklist-90: *r* = 0.74
Trauma	Primary Care PTSD Screen (PC-PTSD-5) (46, 60)	5	n.a.	n.a.	Criterion validity (60): AUC = 0.941
	Childhood Trauma Questionnaire (CTQ) (47, 61)	28	physical abuse, emotional abuse, sexual abuse, emotional neglect, physical neglect	Cronbach’s α = 0.80-0.89 (47)	Construct validity with PHQ-4 (47): *r* = 0.36-0.40
Quality of life	Short Form Health Survey (SF-12) (48, 49)	12	physical health, mental health	Cronbach’s α =0.89 (physical scale), Cronbach’s α = 0.79 (mental health scale, 2-factor model) (48)	Good validity (48)
Pain	Numerical Rating Scale (NRS-11) (50)	1	n.a.	Test-retest reliability = 0.78 (62)	Criterion validity: (relative responsivity): NRS most responsive compared with VAS, VRS (63)
	Widespread Pain Index (WPI) (51)	19	n.a.	Cronbach’s α = 0.83 (64)	Criterion validity: Overall accuracy 95.9% (64)
Work/Job strain	Work ability Indey (WAI) – first item (52)	1	n.a.	na	na
	Kurzform Effort-Reward Imbalance (ERI-S-10) (53)	10	effort, reward	Cronbach’s α = 0.80 (effort), Cronbach’s α = 0.84 (reward) (65)	Satisfactory criterion validity (65)
	Perceived safety climate (PSC-4) (54)	4	n.a.	Cronbach’s α = 0.91 (66)	Construct validity with PSC-12 (66): *r* = 0.97
Symptom patterns	Perceived Causal Relation Network (PECAN) (55–57)	10	n.a.	n.a.	n.a.

n.a., not applicable; AUC, area under the curve.

### Cohort-specific measures

In addition to the core psychometric battery, cohort-specific questionnaires are administered to capture condition-relevant clinical information (e.g., disease activity, symptom severity, and functional impact). These include validated instruments for pain diagnostics in functional-somatic disorders, bowel disease activity in inflammatory bowel disease, hepatic and renal disease characteristics, oncological history, and neuroimmunological or post-infectious symptom profiles (e.g., ME/CFS-criteria-based assessments and autonomic symptom measures). The selection of instruments reflects the clinical standards and diagnostic requirements of each cohort and is harmonised across sites within a common assessment framework.

### Blood sampling

Upon separate informed consent, three routine clinical blood tubes may be collected as part of cohort enrolment: one SST II serum tube (8.5 ml), one EDTA tube (10 ml), and one PAXgene RNA tube (2.5 ml). Laboratory results are documented in the REDCap database by trained study personnel. Blood samples are stored in the respective site-specific biobanks in accordance with established Standard Operating Procedures.

### Statistical methods and analytic framework

All statistical analyses are pre-specified in this protocol and will be conducted using R (R Foundation for Statistical Computing, Vienna, Austria) and IBM SPSS Statistics following completion of data collection. All scales are scored according to established manuals. Missing data due to non-adherence will be systematically examined by exploring patterns of missingness and their associations with symptom severity. Depending on the extent and nature of missing data, analyses will be based on complete cases or appropriate imputation methods, with all decisions and potential impacts on the findings transparently reported. No formal *a priori* power calculation is performed, which is consistent with the exploratory and hypothesis-generating nature of this multicentre study. Given the observational design and cohort heterogeneity, inferential results will be interpreted with emphasis on effect sizes and confidence intervals rather than statistical significance. All analyses are intended to provide a statistical foundation for future confirmatory, longitudinal, and interventional research within the DZPG (German Center for Mental Health) network.

### Patient and public involvement

Patient and public involvement (PPI) is a central component of the infrastructure of the German Center for Mental Health (DZPG). Individuals with lived experience of mental illness, as well as their relatives, contribute experiential knowledge and perspectives that are of high relevance to mental health research. Within the DZPG framework, experts by experience are actively involved in governance structures and contribute to the development and prioritisation of research topics, including those relevant to fatigue and psychosomatic symptom burden. The broader public is engaged, for example, in identifying priorities for future mental health research. For the present study, consultation with patients with lived experience took place during the preparatory phase. Patient representatives were informed about the study aims and overall design and were invited to provide feedback based on their lived experience with fatigue and related psychosomatic symptoms. This consultation informed the refinement of study materials and supported consideration of participant burden and outcome relevance.

### Recruitment and consent

Recruitment takes place at outpatient clinics and specialised consultations corresponding to the seven diagnostic cohorts (see **Table 3** for an overview). Each participating site recruits potential participants from relevant clinical units with the support of attending physicians, psychologists, and research staff. Individuals are informed about the study through flyers and posters displayed at participating clinics and through information provided during routine medical appointments. Depending on the local setting, recruitment may occur in person during clinical visits or via contact initiated by interested individuals through email or telephone. Enrollment is site-specific in this multicentre study. Following provision of detailed written study information and written informed consent, data collection is initiated. The time interval between study enrollment and completion of the questionnaires may vary across participants and sites, as questionnaires may be completed either immediately on site (e.g., during waiting times in outpatient clinics) or subsequently at home. However, in most cases, participants are expected to begin questionnaire completion without substantial delay after enrollment. Eligible individuals who express interest receive detailed written study information, and written informed consent is obtained prior to participation in any study-related procedures.

**Table 3:** Recruitment pathways and inclusion and exclusion criteria for the different study cohorts.

Study cohort	Recruitment site	Recruitment path	Inclusion criteria	Key exclusions
Chronic Primary Pain (CPP)	University Hospital Heidelberg, Department of Psychosomatic Medicine and Psychotherapy; University Hospital Ulm	Rheumatology and psychosomatic consultations	Clinically diagnosed chronic pain disorder	Cancer in the medical history; insufficient German; pregnancy; lactation
Multiple sclerosis	Technical University of Munich, Department and Policlinic of Psychosomatic Medicine and Psychotherapy (Klinikum rechts der Isar)	Multiple sclerosis outpatient clinic	Confirmed MS diagnosis according to McDonald criteria (2017)	Cancer in the medical history; insufficient German; pregnancy; lactation; evidence of current relapse (< 30 days)
Cancer	University Hospital Tübingen, Department of Psychosomatic Medicine and Psychotherapy, in collaboration with the Comprehensive Cancer Center Tübingen-Stuttgart	Psycho-oncology consultation	Confirmed diagnosis of a malignant tumour	Insufficient German; pregnancy; lactation
Chronic renal failure	Charité – Universitätsmedizin Berlin, Department of Psychosomatic Medicine; University Hospital Heidelberg, Department of Psychosomatic Medicine and Psychotherapy	Nephrology outpatient clinics	Ongoing dialysis treatment	Cancer in the medical history; insufficient German; pregnancy; lactation
Inflammatory bowel disease (IBD)	University Hospital Magdeburg, Department of Psychosomatic Medicine and Psychotherapy	Gastroenterology outpatient department (IBD consultation)	Ulcerative colitis or Crohn’s disease	Cancer in the medical history; insufficient German; pregnancy; lactation
Post-COVID syndrome	University Hospital Tübingen, Department of Psychosomatic Medicine and Psychotherapy	Post-COVID outpatient clinic	Diagnosis of Post-COVID syndrome (≥ 1 symptom ≥ 12 weeks after infection)	Cancer in the medical history; insufficient German; pregnancy; lactation
Chronic fatigue syndrome (CFS)	CFS consultation, Department of Infectious Diseases, Charité Berlin	CFS specialty consultation and cross-site recruitment	Diagnosis according to Canadian Consensus Criteria (2003)	Cancer in the medical history; insufficient German; pregnancy; lactation

### Cohorts and eligibility criteria

The study comprises seven diagnostic cohorts representing distinct manifestations of physical–mental interplay across chronic health conditions. These cohorts were selected to capture a broad spectrum of fatigue presentations, ranging from primarily somatic and metabolic to inflammatory, neuroimmunological, post-infectious, and functional–psychosomatic profiles. This design allows comparison of shared and condition-specific patterns of fatigue across different biological and psychosocial constellations.

For conditions with established disease-specific activity markers (e.g. inflammatory parameters, relapse indicators, or clinical staging), relevant clinical information may be extracted from patients’ medical records, where available and where consent has been provided, to contextualise fatigue severity and symptom course. In addition to disease-specific eligibility criteria, the psychometric assessment battery includes measures of depressive symptoms and other comorbidities (e.g., PHQ-9), enabling subsequent analyses to explicitly model their potential effects rather than exclude participants *a priori*. This approach was chosen deliberately to increase ecological validity and to avoid introducing artificial between-cohort differences that may arise from diagnosis-specific exclusion criteria.

Eligible participants are adults aged 18 to 65 years with sufficient German language proficiency and the capacity to provide informed consent. Exclusion criteria include pregnancy, breastfeeding, and insufficient language skills. Detailed inclusion and exclusion criteria, together with the recruiting sites for each diagnostic cohort, are summarised in **Table 3**.

### Recruitment procedures

Recruitment is conducted in a multicentre and cohort-specific manner, following a harmonised framework across all participating sites of the German Center for Mental Health (DZPG). Recruitment procedures are standardised at the level of study information, consent, and eligibility criteria, while logistical aspects are adapted to the clinical context of each cohort and study site. All inclusion and exclusion criteria are applied consistently in accordance with the study protocol.

Participants are informed about the study during routine outpatient care contacts at the collaborating university hospitals. Study information is provided through flyers and posters displayed at participating clinics and through verbal or written information offered during regular clinical encounters. Individuals who express interest are given detailed study information and may decide voluntarily whether to participate.

For analytical purposes, diagnostic cohorts are grouped into four categories reflecting different patterns of physical–mental interplay: metabolic and systemic conditions, inflammatory and oncological conditions, neuroimmunological and post-infectious fatigue syndromes, and chronic primary pain conditions.

### Metabolic and systemic conditions

Participants with chronic renal failure are recruited through specialised outpatient services such as nephrology clinics or dialysis centres. Information about the study is provided during routine clinical visits. Participation may be influenced by treatment schedules and symptom burden; therefore, flexible timing and support for questionnaire completion are offered. In some cases, incomplete online assessments may occur due to health-related constraints.

### Inflammatory and oncological conditions

Participants with inflammatory bowel disease or cancer are recruited through gastroenterology and oncology outpatient clinics. Study information is provided during routine medical appointments and through informational materials displayed at the respective sites. Collaboration between treating physicians, psychosomatic consultants, and research staff supports consistent eligibility screening and facilitates access to study information without exerting recruitment pressure.

### Neuroimmunological and post-infectious fatigue syndromes

This group includes participants with multiple sclerosis, post-COVID syndrome, and myalgic encephalomyelitis/chronic fatigue syndrome (ME/CFS). Recruitment takes place in neuroimmunology and specialised fatigue clinics. Given the fluctuating nature of fatigue severity and daily functioning in these conditions, participants are offered flexible scheduling options and the possibility of remote questionnaire completion to minimise burden.

### Chronic primary pain conditions

Participants with chronic primary pain are recruited through psychosomatic specialty and liaison clinics. Study information is provided during routine consultations, and interested individuals may choose to participate. Where needed, on-site assistance is available to support questionnaire completion, particularly for participants experiencing difficulties with digital data entry.

Across all sites, participation is entirely voluntary and based on written informed consent obtained prior to participation in any study-related procedures. Data are collected via secure survey links and stored in the REDCap database (40, 41) hosted at the University Hospital Tübingen. Recruitment commenced in 2024 and is ongoing across participating sites.

### Variables to be used in analysis

The analytic dataset comprises psychometric, clinical, and sociodemographic variables derived from self-report instruments and, where applicable, from patient records. The core study variables relate to the psychometric characterisation of fatigue across physical, cognitive, and emotional dimensions, assessed using validated questionnaires including the Visual analogue scale (VAS) (22), Chalder Fatigue Scale (24), DePaul Symptom Questionnaire Post-Exertional Malaise (27), Patient-Reported Outcomes Measurement Information System (PROMIS Fatigue) (31), Systemic Exertion Intolerance Disease (SEID) criteria of the Institute of Medicine (33), and the Short Form (SF-12) Health Survey – Physical Functioning subscale (SF-36) (36).

In addition, a predefined set of associated variables is included to capture mental-health symptoms, somatic symptom burden, and psychosocial context factors. These include depressive symptoms (Patient Health Questionnaire, PHQ-9 (42)), anxiety (Generalised Anxiety Disorder Scale, GAD-7), somatisation (PHQ-15 (44)), psychological responses to bodily symptoms (SSD-12 (45)), trauma exposure (Primary Care PTSD Screen, PC-PTSD-5 (46) and Childhood Trauma Questionnaire, CTQ (47)), quality of life (Short Form Health Survey, SF-12 (48, 49)), pain (Numerical Rating Scale, NRS (50); Widespread Pain Index, WPI (51)), occupational functioning (Work Ability Index, WAI (52)), and psychosocial work environment (Effort-Reward Imbalance, ERI-Short Form (53); Psychosocial Safety Climate, PSC-4 (54)).

Sociodemographic data (e.g. age, sex, education, employment status) and relevant medical information (diagnosis, comorbidities, and disease activity markers where available) are collected to enable adjustment for confounding and subgroup analyses. A comprehensive overview of all psychometric instruments, including their theoretical framework, operationalisation, scoring structure, response format, psychometric properties, and corresponding references, is provided in **Tables 1**, **2**. The table summarises each construct assessed in the study covering the respective instrument name, citation, subscales and number of items, response format, conceptual focus, analytic approach (including cut-off values where applicable), and available reliability and validity indices. Together, these details ensure full transparency and reproducibility of the measurement framework. Additionally, where participants provided consent, self-reported mental and other clinical health outcomes may be complemented by information extracted from their medical records to support contextual interpretation of fatigue severity and associated symptom burden.

## Discussion

The present study based on a multicentre design provides a unique opportunity to characterise fatigue across a wide range of physical and mental health conditions using an extensive assessment battery of fatigue and mental health masures. By including multiple clinical cohorts within a single methodological framework, this study addresses a central limitation of much of the existing fatigue literature, which has largely focused on disorder-specific samples and has rarely allowed direct cross-condition comparisons.

Although the psychometric assessment battery employed in this study is extensive, its scope reflects a deliberate methodological decision. Fatigue is a multidimensional construct shaped by interacting physical, cognitive, emotional, psychosocial, and contextual factors. Capturing this complexity requires the parallel assessment of multiple symptom domains and related influences. The breadth of instruments therefore represents a key strength of the study, enabling a level of phenotypic resolution that is rarely achieved in fatigue research and providing a solid empirical basis for transdiagnostic comparisons.

To ensure feasibility for participants, particularly those experiencing pronounced fatigue, several measures were implemented to reduce participation burden. These include on-site technical support, the option for personal assistance by trained study staff, and the possibility to pause and resume the online assessment. Recruitment within clinical settings was intended to lower participation barriers and facilitate access to study-related information and support. While incomplete assessments or delayed completion may occur in this context, these will be addressed analytically through planned sensitivity analyses and appropriate handling of missing data.

From a methodological perspective, ecological momentary assessment (EMA, (19, 67)) represents a promising approach for capturing short-term symptom dynamics and contextual influences on fatigue in daily life. In contrast to cross-sectional assessments, EMA can provide detailed information on intra- and interindividual variability, including fluctuations within and between days, which may be particularly relevant for understanding fatigue patterns across different disease populations. However, EMA protocols can be associated with high-frequency, repeated assessments and require substantial temporal and cognitive engagement from participants. As the primary aim of the present study was to include and compare different fatigue questionnaires across cohorts, a longitudinal EMA-based assessment would have posed an excessive cognitive burden for patients experiencing fatigue symptoms. To this end, a comprehensive and harmonised questionnaire battery was deliberately prioritised to assess multiple fatigue dimensions alongside relevant psychological, somatic, and psychosocial correlates. Combining this already extensive assessment with additional EMA protocols would have substantially increased the complexity of data collection and potentially limited comparability across cohorts and study sites in a large multicentre design.

Accordingly, a cross-sectional, questionnaire-based approach was selected as a pragmatic and methodologically coherent first step. Building on the results of this study, future research may employ more targeted designs, including EMA-based or hybrid approaches, to examine temporal dynamics, situational influences, and intraindividual variability of fatigue in selected subgroups or specific clinical contexts.

Overall, the systematic breadth of assessment combined with a pragmatic, participant-oriented recruitment strategy positions this study to advance understanding of fatigue as a multidimensional and transdiagnostic phenomenon. The findings are intended to inform future longitudinal and interventional research aimed at elucidating underlying mechanisms and supporting the development of tailored approaches to fatigue across diverse physical and mental health conditions.
